# High-dose concurrent chemo–proton therapy for Stage III NSCLC: preliminary results of a Phase II study

**DOI:** 10.1093/jrr/rru034

**Published:** 2014-05-25

**Authors:** Yoshiko Oshiro, Toshiyuki Okumura, Koichi Kurishima, Shinsuke Homma, Masashi Mizumoto, Hitoshi Ishikawa, Masataka Onizuka, Mitsuaki Sakai, Yukinobu Goto, Nobuyuki Hizawa, Yukio Sato, Hideyuki Sakurai

**Affiliations:** 1 Department of Radiation Oncology, University of Tsukuba, Tsukuba, Ibaraki, Japan; 2 Department of Respiratory Medicine, University of Tsukuba, Tsukuba, Ibaraki, Japan; 3 Department of Respiratory Surgery, University of Tsukuba, Tsukuba, Ibaraki, Japan

**Keywords:** proton therapy, radiotherapy, lung cancer, Phase II study, chemo–proton therapy

## Abstract

The aim of this report is to present the preliminary results of a Phase II study of high-dose (74 Gy RBE) proton beam therapy (PBT) with concurrent chemotherapy for unresectable locally advanced non-small-cell lung cancer (NSCLC). Patients were treated with PBT and chemotherapy with monthly cisplatin (on Day 1) and vinorelbine (on Days 1 and 8). The treatment doses were 74 Gy RBE for the primary site and 66 Gy RBE for the lymph nodes without elective lymph nodes. Adapted planning was made during the treatment. A total of 15 patients with Stage III NSCLC (IIIA: 4, IIIB: 11) were evaluated in this study. The median follow-up period was 21.7 months. None of the patients experienced Grade 4 or 5 non-hematologic toxicities. Acute pneumonitis was observed in three patients (Grade 1 in one, and Grade 3 in two), but Grade 3 pneumonitis was considered to be non-proton-related. Grade 3 acute esophagitis and dermatitis were observed in one and two patients, respectively. Severe ( ≥ Grade 3) leukocytopenia, neutropenia and thrombocytopenia were observed in 10 patients, seven patients and one patient, respectively. Late radiation Grades 2 and 3 pneumonitis was observed in one patient each. Six patients (40%) experienced local recurrence at the primary site and were treated with 74 Gy RBE. Disease progression was observed in 11 patients. The mean survival time was 26.7 months. We concluded that high-dose PBT with concurrent chemotherapy is safe to use in the treatment of unresectable Stage III NSCLC.

## INTRODUCTION

The prognosis of unresectable advanced non-small-cell lung cancer (NSCLC) remains poor despite advances in radiotherapy and medication. Concurrent chemoradiotherapy is the first treatment choice for unresectable advanced NSCLC. In the 2000s, dose escalation studies were encouraged, and doses > 70 Gy were delivered with concurrent chemotherapy [1–6]. The prognoses were favorable in many Phase I/II studies, with median survivals of > 20 months and toxicities that appeared tolerable [3–6]. However, in the Phase III study, there was no apparent survival benefit [7]. While the reason was unclear, cardiopulmonary toxicities were suspected as potential contributors [8]. Proton beam therapy (PBT) has been utilized in advanced lung cancer [9–11]. Proton beams can reduce the doses for normal lung tissues because of the penetration energy peak, the ‘Bragg peak’. Therefore, we hypothesized that high-dose PBT with concurrent chemotherapy might be well tolerated and lead to favorable results. We initiated a Phase II study in 2010 to evaluate the safety and efficacy of high-dose PBT with concurrent chemotherapy for unresectable or medically inoperable advanced NSCLC. Herein, we report the preliminary results.

## MATERIALS AND METHODS

### Patients

This Phase II study was approved by the ethics board of Tsukuba University, and written informed consent was obtained from each patient. Patients with unresectable or medically inoperable, histologically or cytologically confirmed Stage II and Stage III NSCLC (according to the TNM classification of malignant tumors, 7th edition) were enrolled between February 2010 and January 2013. Disease in all cases was staged using computed tomography (CT) of the chest and abdomen, magnetic resonance imaging (MRI) of the brain, and bone scintigram one month prior to enrollment. 2-[18F]-fluoro-2-deoxy-D-glucose-positron emission tomography (FDG-PET) was not essential in this study. Other eligibility criteria included age between 20 and 70 years, performance status (PS) of 0–1, adequate principal organ function with serum white blood cell count (WBCs) ≥ 3000/μl, neutrophil cells (NTRs) ≥ 1500/μl, platelets (PLTs) ≥ 100 000/μl, hemoglobin (Hb) ≥ 9.0 g/dl, serum creatinine (Cre) ≤ 1.2 mg/dl, creatinine clearance (Ccr) ≥ 60 ml/min (according to the Cockcroft–Gault equation), serum alanine aminotransferase (ALT) < 100 U/l, aspartate amino transferase (AST) < 100 U/l, total bilirubin (T-Bil) < 1.5 mg/dl, forced expiratory volume after 1.0 s (FEV1.0) ≥ 0.75 l, and arterial oxygen pressure (PaO_2_) ≥ 60 Torr.

Patients with contralateral hilar lymph node metastasis, intrapulmonary metastasis in the same lobe, obvious interstitial pneumonitis on imaging, or uncontrollable hypertension and diabetes were excluded. Patients who had undergone thoracic radiotherapy or chemotherapy in the past five years, patients with lung cancer within the past two years and those with a malignant tumor at another site were also excluded.

### Proton beam therapy

For treatment planning, chest CT images were obtained in 5-mm thick slices in the treatment position, with a respiratory-gated system during the end-expiratory phase. The gross target volume (GTV) was defined as the primary tumor and clinically positive lymph nodes. The clinical target volume (CTV)-1 encompassed the primary tumor and the locoregional lymph nodes where clinically positive lymph nodes existed. Prophylactic lymph nodes were not included. Clinically positive lymph nodes were defined as nodes ≥ 1 cm on a CT scan or as PET-positive lymph nodes. The planned target volume (PTV)-1 covered the CTV-1 with a 7–10-mm margin in all directions and an additional 5-mm margin in the caudal direction to compensate for respiratory motion. CTV-2 was defined as only the primary tumor, and PTV-2 was settled as well. The treatment doses of 74 Gy RBE in 37 fractions and 66 Gy RBE in 33 fractions were delivered to PTV-2 and PTV-1, respectively. The targets were delineated as maximal contour on the lung and mediastinum window. The RBE of the proton beam was assigned a value of 1.1 [12]. Adapted planning was made with reduction in tumor volume.

Treatment beams were delivered during the end-expiratory phase using a respiratory gating system controlled by a laser range finder that monitors the movement of the patient's body surface. The patient's body was immobilized using a custom-shaped body cast (ESFORM, Engineering System Co., Matsumoto). Prior to each treatment, the patient's position was confirmed by fluoroscopy.

The termination criteria of PBT were as follows: WBCs < 1000/μl, NTRs < 500/μl, PLTs < 5000, fever ≥ 38°C. PBT was stopped when radiation pneumonitis was observed, and the patients were withdrawn from this study when PBT could not be reinitiated within 14 d, or disease progression was observed.

### Chemotherapy

All patients received monthly concurrent cisplatin (CDDP) and vinorelbine (VNR) as intravenous infusions during PBT. CDDP was administered at 80 mg/m^2^ on Day 1 and VNR was administered at 20 mg/m^2^ on Days 1 and 8. The two courses of chemotherapy were administered during PBT. While neoadjuvant (induction) chemotherapy was not allowed, adjuvant (consolidation) was allowed in this study.

The discontinuance criteria of VNR on Day 8 were as follows: WBCs < 3000/μl, NTRs < 1500/μl, PLTs < 100 000/μl, infectious fever up ≥ 38°, ALT ≥ 100 U/l, AST ≥ 100 U/l, T-Bil ≥ 1.5 mg/dl, and non-hematologic toxicity ≥ Grade 3.

The continuance criteria for the second course were: WBCs ≥ 3000/μl, NTRs ≥ 1500/μl, PLTs ≥ 100 000/μl, Cre ≤ 1.2 mg/dl, Ccr ≥ 60 ml/min, ALT < 100 U/l, AST < 100 U/l, T-Bil < 1.5 mg/dl and PS ≤ 1. The second course of CDDP and VNR was reduced to 60 mg/m^2^ and 15 mg/m^2^, respec-tively, when VNR was skipped during the first course, or toxicities were observed during the first course, as follows: WBCs < 1000/μl, NTRs < 500/μl, PLTs < 25 000/μl, Cre > 1.6 mg/dl and non-hematologic toxicity ≥ Grade 3. Chemotherapy was stopped when the tumor progressed, severe (≥ Grade 3) toxicities of pneumonitis, kidney, liver or peripheral nerve were observed, if the second course could not be initiated within 14 d of the scheduled date, or if the patient refused chemotherapy.

### Follow-up

Patients were evaluated at least weekly during treatment, and every 2–3 months after the PBT for 1 year, and 3–6 months, thereafter. Acute and late toxicities were defined as evaluated and graded according to the Common Terminology Criteria for Adverse Events (CTCAE) version 3 [13]. Acute toxicities were defined as occurring during and within 6 months after the chemo–proton therapy, and late toxicities were defined as those appeared 6 months after the completion of chemo–proton therapy. The survival and recurrence were calculated from the date of the start of chemoradiotherapy.

The response rate was evaluated and classified into complete response (CR), partial response (PR), progressive disease (PD) and stable disease (SD), according to the modifications of the Response Evaluation Criteria in Solid Tumors (RECIST) [14]. Local recurrence was defined as an increase in tumor size > 20%, or significant positive accumulation on the PET imaging.

### Statistical analysis

The primary endpoint was toxicity, and the secondary endpoints were overall survival, progression-free survival, local control rate and response rate. The survival was analysed using the Kaplan–Meier method (SPSS, IBM Inc., NY, USA).

## RESULTS

A total of 17 patients were enrolled in this study, and two patients were withdrawn. Obstructive pneumonia could not be controlled in one patient, and the chemotherapy agents were changed before the start of treatment. The other patient could not continue with the PBT because of the Great East Japan Earthquake, and photon radiotherapy was used as an alternative. Therefore, 15 patients were evaluated in this study.

The characteristics of the 15 patients are presented in Table 1. Four and 11 patients had Stage IIIA and III B disease, respectively, and all patients had unresectable NSCLC. The median CTV volume was 191.3 cm^3^.

**Table 1.** Patient characteristics

**Age**	
Median (range)	60 (40–68)
**Sex**	
Male	13
Female	2
**Stage**	
II	0
IIIA	4
IIIB	11
**Pathology**	
Adenocarcinoma	7
Squamous cell carcinoma	5
Non-small-cell carcinoma	2
Adenoidcystic carcinoma	1
**Clinical target volume**	
Median (range) (cc)	191.3 (33.1–817.3)
**Status**	
Alive	9
Dead	6
**Local recurrence**	
Yes	6
No	9

**Fig. 1.**
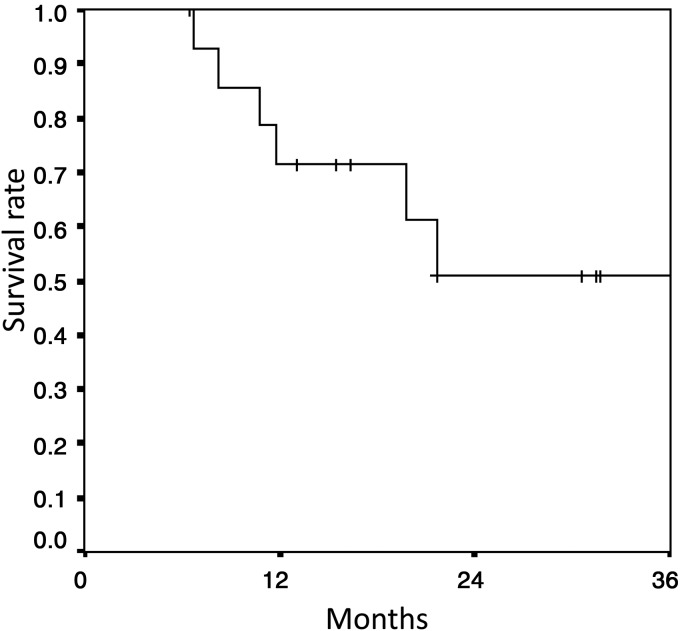
Overall survival of patients.

**Fig. 2.**
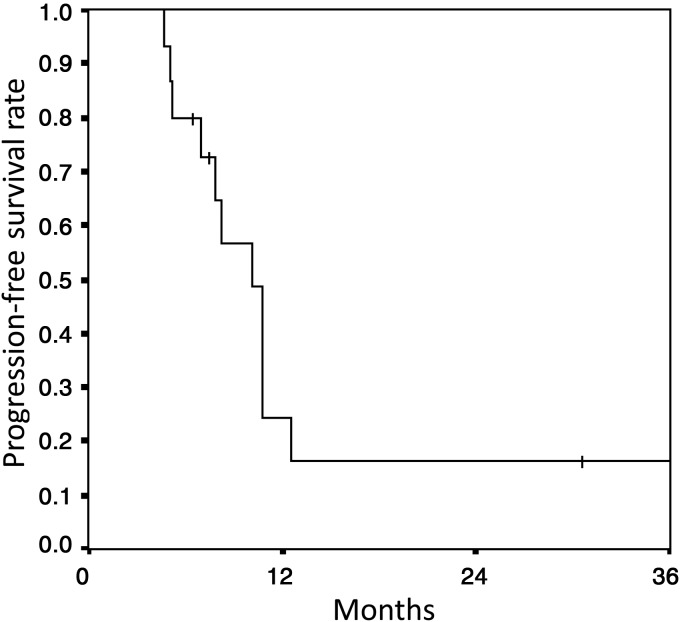
Progression-free survival of patients.

At the time of analysis, nine patients were alive. The median follow-up period for the survivors was 21.7 months (range: 7–39 months). The mean survival time was 26.7 months (95% confidence interval (CI): 19.5–33.9 months), and the 2-year overall survival time was 51% (95% CI: 21.7–80.3%) (Fig. 1). The median progression-free survival was 10.2 months (95% CI: 8.0–12.4 months), and the 1- and 2-year progression-free survival rates were 24.2% (95% CI: 1.0–48%) and 16.1% (95% CI: 0–36.6%), respectively (Fig. 2). The acute toxicities are presented in Table 2. Among the non-hematologic acute toxicities, Grade 3 pneumonitis was observed in two patients and was diagnosed as infectious pneumonia and obstructive pneumonia on the basis of clinical course and image findings. No severe radiation pneumonitis was observed. Grade 3 esophagitis and skin reactions were observed in one and two patients, respectively. Among the hematologic toxicities, severe ( ≥ Grade 3) leukocytopenia, neutropenia and thrombocytopenia were observed in 10 patients, 7 patients and 1 patient, respectively. In particular, Grade 4 neutropenia was observed in four patients (26.7%). The full doses of the first and second courses of chemotherapy were completed in 13 (87%) and eight (53%) patients, respectively.

**Table 2.** Acute toxicities

	Toxicity grade					
	0	1	2	3	4	5
**Bone Marrow**						
Leukocytopenia	1	2	2	10	0	0
Neutropenia	2	1	5	3	4	0
Hemoglobin reduction	1	12	2	0	0	0
Thrombocytopenia	13	1	0	1	0	0
**Lung**						
Cough	14	1	0	0	0	0
Pneumonitis	12	1	0	2^a^	0	0
Dyspnea	14	1	0	0	0	0
**Gastrointestinal**						
Appetite loss	13	2	0	0	0	0
Nausea	14	1	0	0	0	0
Esophagitis	7	3	4	1	0	0
Weight loss	14	0	1	0	0	0
**Other**						
Dermatitis	4	4	5	2	0	0
Hyperbilirubinemia	14	1	0	0	0	0
Singultation	14	1	0	0	0	0

^a^Infectious pneumonitis and obstructive pneumonia were suspected according to imaging and clinical course.

Late toxicities were evaluated in 13 patients. One patient experienced Grade 3 radiation pneumonitis, and another patient experienced Grade 2 radiation pneumonitis. Grade 1 vasculitis and skin atrophy were also observed in one patient each.

The tumor response upon completion of PBT was PR in 6 and SD in 9. Disease progression was observed in 11 patients during the follow-up period. The first progression site was local in six patients, bone metastasis in two patients, and brain, intrapulmonary and lymph nodes outside the irradiation field in one patient each. All of the local recurrences occurred in the primary tumors, not in the lymph nodes. The course of each patient is shown in Table 3.

**Table 3.** Clinical course of each patient

No.	Stage	CTV (cm^3^)	#1 CDDP + VNR	#2 CDDP + VNR	Local effects	Adjuvant (#3, 4) chemo	Local recurrence	Progression	Treatment for recurrences	Overall survival (M)	Cause of death
1	IIIB	222.2	VNR skip	Reduced	SD	No	Yes	Local	chemo	19.8	Cancer
2	IIIB	65.7	Full	skip	SD	Yes	No	None	-	38.8	Alive
3	IIIA	93.9	Full	Reduced	PR	No	Yes	Marginal & Local	none	21.7	Cancer
4	IIIA	478.8	Full	skip	PR	No	Yes	Local	chemo	11.8	Cancer
5	IIIB	537.1	VNR skip	Reduced	SD	No	Yes	Local	none	10.8	Cancer
6	IIIB	155.8	Full	Reduced	PR	No	No	None	-	30.5	Alive
7	IIIB	192.6	Full	Full	PR	Yes	No	Lymph nodes	chemo, RT^#^	21.7	Alive
8	IIIB	167.1	Full	Full	PR	Yes	No	Brain	chemo RT	31.4	Alive
9	IIIB	376.2	Full	Full	SD	Yes	Yes	Local	chemo RT	31.7	Alive
10	IIIB	190.0	Full	Full	SD	Yes	No	Bone	RT	6.7	Cancer
11	IIIA	185.1	Full	Full	SD	Yes	No	None	-	8.2	CVD^||^
12	IIIB	817.3	Full	Reduced	SD	No	No	Intrapulmonary	chemo	13.1	Alive
13	IIIB	389.7	Full	VNR skip	PR	Yes	Yes	Local	chemo	15.5	Alive
14	IIIB	33.1	Full	Full	SD	Yes	No	Bone	chemo	16.3	Alive
15	IIIA	170.7	Full	Full	SD	No	No	None	-	6.5	Alive

CTV = clinical target volume, CDDP = cisplatin, VNR = vinorelbine, SD = stable disease, PR = partial response, chemo = chemotherapy including molecularly targeted drug, RT = radiotherapy, CVD = cerebrovascular disease.

## DISCUSSION

Dose escalation with concurrent chemoradiotherapy for advanced NSCLC is controversial. The median survival time was 16–26 months in Phase I/II studies of high-dose radiotherapy in combination with concurrent chemotherapy [1–6]. However, in a Phase III study, the one-year survival rate was 70.4% in the 74 Gy arm, which was much inferior to the 60 Gy arm (80%) [7]. The reason for the negative results in the Phase III study is not clear. However, Cox *et al.* suggested the contribution of cardiopulmonary toxicities [8].

Excellent dose localization of proton beams can reduce normal tissue doses. Roelofs *et al*. suggested that PBT gave the lowest dose to organs at risk (lung, esophagus, spinal cord and heart) compared with 3D conformal radiotherapy and intensity-modulated radiotherapy, while maintaining doses of 70 Gy to the target [15]. Nichols *et al*. evaluated the dose distribution in eight patients with Stage III disease and found that PBT led to about a 30% reduction of the normal lung volume receiving 20 Gy (V_20_) in all patients [16]. Therefore, reduction of the toxicity is anticipated by the use of proton beams, even though high doses are delivered to the tumor. However, there have been few reports of high-dose chemo–proton therapy [17, 18]. Recently, Chang *et al*. reported a Phase II study with high-dose chemo–proton therapy using 74 Gy RBE and weekly carboplatine and paclitaxel. In this study, the median survival period was 29.4 months and the toxicities were considered acceptable [17].

In our series, CDDP and VNR were used as a chemotherapy regimen, according to the studies by the Cancer and Leukemia Group B (CALGB) [19] and Sekine *et al*. [20]. In the Phase II study by CALGB, gemcitabine (GEM), PTX and VNR were compared as additional agents used with CDDP for concurrent chemoradiotherapy at a dose of 66 Gy. There was a significant difference in median survival time (MST) between the three agents. However, esophagitis and platelet depletion and granulocytopenia occurred frequently in the GEM and PTX groups, respectively [19]. Sekine *et al*. reported the MST of 30.4 months for unresectable Stage III NSCLC treated concurrently with 60 Gy of photon radiotherapy and CDDP and VNR chemotherapy [20]. Therefore, we conducted chemoradiotherapy with CDDP and VNR, as performed previously (unpublished data), and continued this regimen. We found that radio-toxicities were relatively mild and well tolerated using PBT of 74 Gy with CDDP and VNR. However, nearly half of the patients were unable to complete chemotherapy, and myelosuppression occurred frequently under this chemotherapy regimen.

Meanwhile, local recurrence in the primary lesion was observed in six patients (40%) in our series. This occurred more often than the finding of 5% reported by Hoppe *et al*. [18] and of 20.5% reported by Chang *et al*. [17], and this may be due to the fact that the tumors were large and at an advanced stage (IIIB disease in the most patients) in our study with a mean CTV of 191.3 cm^3^ (almost twice the CTV reported by Chang *et al*. (median: 101.3 cm^3^)). Of the 19 patients in the study by Hoppe *et al*., 16 had Stage IIIA disease [18]. In addition, Chang *et al*. set CTVs as the GTVs (defined as maximum image verified across all phases of the 4D CT) plus 8 mm margins [17]. Tumor size may have been assessed differently because additional uniform CTV margins were not added to the GTVs on the CT images obtained at the expiratory phase in our study. In five of the six patients who experienced local recurrence the CTV was > 222 cm^3^. The one patient with a CTV of 99 cm^3^ exhibited marginal recurrence. In contrast, there was no in-field lymph node recurrence, even though 66 Gy RBE was delivered in this region. Therefore, it would appear that while 66 Gy RBE was sufficient to control lymph nodes, there are limitations to its ability to control large tumors, even with the high dose of 74 Gy RBE.

Because of the short follow-up period, survival has not been assessed to date. However, the current mean survival time of 26.7 months is comparable with previous reports of Phase I/II high-dose concurrent chemoradiotherapy. Most of our patients experienced disease progression, and many received adjuvant therapy, including chemotherapy, molecular-targeted agents, and photon radiotherapy. We consider that improvements in survival for advanced NSCLC could be achieved by multimodality therapy, and our study suggests that 74 Gy RBE of PBT with concurrent CDDP and VNR is safe and useful in the multimodality therapy for unresectable NSCLC.

## FUNDING

This work was supported in part by Grants-in-Aid for Scientific Research (B) (24390286) from the Ministry of Education, Science, Sports and Culture of Japan. Funding to pay the Open Access publication charges for this article was provided by Grants-in-Aid for Scientific Research (B) (24390286) from the Ministry of Education, Science, Sports and Culture of Japan.
